# Deleterious Germline *BLM* Mutations and the Risk for Early-onset Colorectal Cancer

**DOI:** 10.1038/srep14060

**Published:** 2015-09-11

**Authors:** Richarda M. de Voer, Marc-Manuel Hahn, Arjen R. Mensenkamp, Alexander Hoischen, Christian Gilissen, Arjen Henkes, Liesbeth Spruijt, Wendy A. van Zelst-Stams, C. Marleen Kets, Eugene T. Verwiel, Iris D. Nagtegaal, Hans K. Schackert, Ad Geurts van Kessel, Nicoline Hoogerbrugge, Marjolijn J.L. Ligtenberg, Roland P. Kuiper

**Affiliations:** 1 Department of Human Genetics, Radboud university medical center, Geert Grooteplein 10, 6525 GA, Nijmegen, The Netherlands; 2 Department of Pathology, Radboud university medical center, Geert Grooteplein 10, 6525 GA, Nijmegen, The Netherlands; 3 Department of Surgical Research, University Hospital Carl Gustav Carus, Technische Universität Dresden, Fetscherstraße 74, 01307, Dresden, Germany

## Abstract

Bloom syndrome is an autosomal recessive disorder characterized by chromosomal instability and increased cancer risk, caused by biallelic mutations in the *RECQL*-helicase gene *BLM*. Previous studies have led to conflicting conclusions as to whether carriers of heterozygous *BLM* mutations have an increased risk to develop colorectal cancer (CRC). We recently identified two carriers of a pathogenic *BLM* mutation in a cohort of 55 early-onset CRC patients (≤45 years of age), suggesting an overrepresentation compared to the normal population. Here, we performed targeted sequencing using molecular inversion probes to screen an additional cohort of 185 CRC patients (≤50 years of age) and 532 population-matched controls for deleterious *BLM* mutations. In total, we identified three additional CRC patients (1.6%) and one control individual (0.2%) that carried a known pathogenic *BLM* mutation, suggesting that these mutations are enriched in early-onset CRC patients (*P* = 0.05516). A comparison with local and publically available databases from individuals without suspicion for hereditary cancer confirmed this enrichment (*P* = 0.003534). Analysis of family members of the five *BLM* mutation carriers with CRC suggests an incomplete penetrance for CRC development. Therefore, these data indicate that carriers of deleterious *BLM* mutations are at increased risk to develop CRC, albeit with a moderate-to-low penetrance.

In autosomal recessive cancer predisposing syndromes such as Bloom syndrome (MIM2109000), Nijmegen breakage syndrome (MIM251260), and Fanconi anemia (MIM227650), the risk for cancer is age-dependent and varies between cancer types. Currently, evidence accumulates that also monoallelic mutations in the genes associated with these autosomal recessive syndromes, *BLM*, *NBN* and *FANCC*, respectively, may result in increased risks for cancer^1^,^2^,^3^. Heterozygous deleterious mutations in the DNA *RECQL*-helicase gene *BLM* have been associated with an increased risk for breast cancer^3^, but studies on the role of these mutations in susceptibility to CRC have been conflicting^4^,^5^,^6^. In Bloom syndrome patients, the lifetime risk for developing CRC is approximately 12%^7^. Previous research on heterozygous carriers of *BLM* mutations and CRC risk has mainly focused on the *BLM* founder mutation p.Y736 fs (*BLM*^*Ash*^) in the Ashkenazi Jewish population. Whereas Gruber *et al.* described that Ashkenazi Jews with CRC carry the *BLM*^Ash^ allele more than twice as frequent as matched controls, Cleary *et al.*^5^ were unable to replicate these findings and Baris *et al.*^6^ did not find an increased risk for CRC in *BLM*^*Ash*^ mutation carriers.

Very recently, we performed whole-exome sequencing on a cohort of individuals diagnosed with CRC (*n* = 55) before the age of 45 years, and we identified two individuals with heterozygous *BLM* mutations that are known to play a role in Bloom syndrome (De Voer *et al.* unpublished data). To reveal whether these mutations contribute to the risk for CRC development, we have performed a case-control sequencing study using targeted enrichment by molecular inversion probe technology^8^ followed by Ion semiconductor sequencing to compare the prevalence of Bloom syndrome-causing mutations in patients with CRC and healthy controls.

## Results

### Frequency of *BLM* mutations in the general population

**Figure 1:**
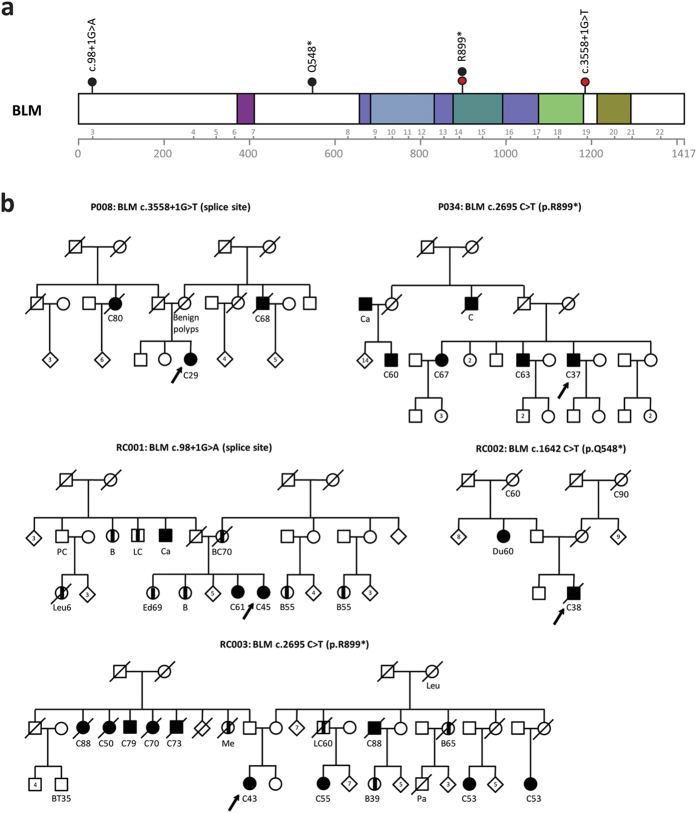
Deleterious mutations in *BLM* in individuals with early-onset CRC. (**a**) Distribution of deleterious mutations identified in the CRC discovery cohort (red dots) and replication cohort (black dots) in *BLM*. Structural domains of the protein are indicated in colours. (**b**) Pedigrees of the individuals with deleterious *BLM* mutations. An arrow indicates the proband. Individuals with cancer and their age at diagnosis (if known) are marked as follows: breast cancer (B), bladder cancer (BC), brain tumour (BT), colon cancer (C), endometrial cancer (Ed), leukaemia (Leu), liver cancer (LC), pancreas cancer (Pa), melanoma (Me), prostate cancer (PC) and cancer of unknown origin (Ca).

We previously identified deleterious *BLM* mutations, a splice site mutation (c.3558 + 1G > T) and a nonsense mutation (c.2695C > T; p.Arg899*), in two individuals that were diagnosed with colorectal cancer at the age of 29 and 37 years, respectively (Fig. 1a and Table 1; deVoer *et al.* unpublished data). Both mutations were reported in the Bloom’s syndrome registry, and are thus found in Bloom’s syndrome patients^9^. Since there are no precise numbers on the frequency of pathogenic *BLM* mutations in the general population, we queried the data browser from the Exome Aggregation Consortium (ExAC; *n* = 61,486)^10^, a publically available dataset containing exomes from individuals of European, African or Asian ancestry. We established an overall prevalence of known deleterious *BLM* mutations of approximately one in 900 individuals (0.11%), suggesting that the prevalence of *BLM* mutations is enriched in our cohort of 55 early-onset CRC patients. To determine whether we could validate these findings, we managed to collect and analyse a cohort of 185 CRC patients diagnosed with CRC before the age of 50 years originating from the Netherlands (*n* = 105) and Germany (*n* = 80), as well as a control cohort of 532 healthy individuals, for the presence of deleterious *BLM* mutations.

### Targeted re-sequencing of *BLM* in cases and controls

For the targeted enrichment of the entire coding sequence of the *BLM* locus, we used molecular inversion probes (MIPs) technology, a recently developed method that facilitates high-throughput, multiplex sequence analyses, with high specificity for the target sequence^8^. The coding sequence of *BLM*, including the intron-exon boundaries, was targeted for 97.4% using 81 MIPs. The untargeted sequences involved two coding regions within exons 10 and 13, which do not overlap with known pathogenic mutations (Supplementary Figure 1). Sequencing of the MIP amplified regions was performed using Ion Torrent PGM sequencing in eight different runs, containing between 89 and 138 barcoded samples per run. On average, 99.2% of the target sequence of the CRC cohort had a coverage of ≥10-fold (95.2% with ≥50-fold coverage), with a median coverage of 745-fold per probe (Supplementary Figure 2A). In the control cohort, on average, 98.7% of the target sequence was covered at least 10-fold (91.3% with ≥50-fold coverage), with a median coverage of 320-fold per probe (Supplementary Figure 2B).

We identified heterozygous deleterious *BLM* mutations in three out of 185 CRC cases: one splice site mutation (c.98 + 1G > A) and two nonsense mutations (c.1642C > T; p.Gln548* and c.2695C > T; p.Arg899* mutation, 1.6%; Fig. 1a and Table 1), and in one out of 532 control samples (c.1642C > T; p.Gln548*, 0.2%), which points towards an enrichment in early-onset CRC cases compared to controls with borderline significance (OR = 8.67; 95% CI, 0.69–455.36; *P* = 0.05516). In addition, we analysed an in-house cohort of individuals without a suspicion for hereditary cancer for which high-quality exome sequencing data were available (*n* = 2,329; median exome coverage ≥90-fold; see materials and methods). Two *BLM* mutations (0.1%) were found to be present in this dataset, which was significantly lower compared to our CRC cohort (*P* = 0.003534; Table 2).

### Second-hit mutation analyses in tumours of *BLM* mutation carriers

**Figure 2:**
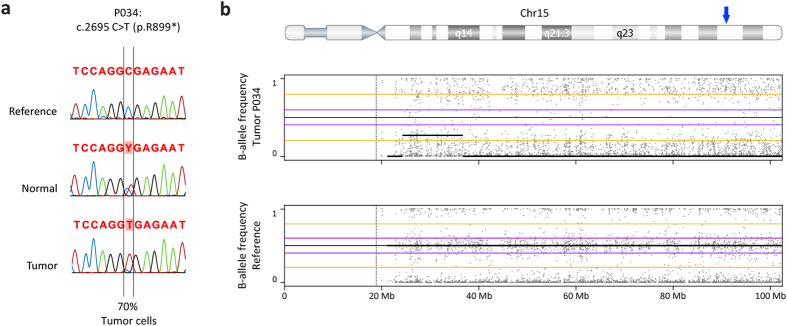
Loss of heterozygosity analyses of the deleterious *BLM* mutation in the tumour of P034. (**a**) Sanger sequencing results of the region around the variants in reference DNA, DNA from normal- and tumour tissue from the patient, showing a loss of the wild-type C-allele in the tumour. (**b**) B-allele frequency plot of chromosome 15 showing the loss of heterozygosity along the entire chromosome 15 in the tumour of individual P034 (top) as compared to a reference (bottom). A blue arrow on the top ideogram marks the *BLM* locus.

*BLM* is thought to be a classical tumour suppressor gene^11^,^12^, suggesting that somatic events targeting the wild-type *BLM* allele may contribute to the development of CRC. Therefore, we analysed four available tumours and matched normal colonic tissue for loss of heterozygosity (LOH) and mutations in the *BLM* locus (Table 1). In DNA from one tumour (P034) a clear reduction of the wild-type allele compared to the normal tissue was observed, suggesting loss of the wild-type *BLM* allele in the tumour (Fig. 2a). Subsequent genome-wide profiling of this tumour showed that multiple regions exhibited copy-number neutral LOH, including the entire q-arm of chromosome 15 on which the *BLM* gene is located (Fig. 2b and Supplementary Figure 2). On the remaining three tumours second-hit mutation analysis was performed using MIP-based sequencing of the entire coding region of *BLM*. However, due to poor quality and low quantity of the DNA this analysis was only successful for one tumour (RC002), which did not reveal additional somatic mutations in the coding region of *BLM*.

### Risk for CRC in heterozygous *BLM* mutation carriers

Next, we collected clinical information from all five CRC patients in which we identified the *BLM* mutations, as well as from members of the corresponding families (Fig. 1b). Several first-degree relatives of index cases P034 and RC001 have a history of CRC, and cases P008, RC002 and RC003 have multiple second-degree relatives with CRC. The affected sister of case RC001 was found to be carrier of a pathogenic *MSH2* mutation (data not shown), which was not present in the *BLM* mutation-positive proband. We were unsuccessful in collecting DNA from the parents of the probands or other family members to perform co-segregation analyses. However, all identified *BLM* mutations have previously been described^9^ and reported in the data from the ExAC browser, strongly suggesting that they are inherited from either one of the parents.

## Discussion

Using exome sequencing we previously identified two individuals with early-onset CRC (≤45 years of age) that carried a deleterious *BLM* mutation in a cohort of 55 CRC cases (de Voer *et al.* unpublished data). We estimated that on average only one in every 900 individuals in the general population is a carrier of a known deleterious *BLM* mutation, indicating an enrichment of *BLM* mutation carriers amongst individuals that develop CRC at an early age. Targeted re-sequencing of the *BLM* locus in an additional cohort of cases and controls confirmed this enrichment in individuals with early-onset CRC. In the tumour of one of these individuals, the *BLM* gene was found to be completely inactivated by somatic loss of the wild-type allele. Analyses of the familial histories for cancer revealed that carriers of a heterozygous deleterious *BLM* mutation most likely have a low-to-moderate penetrant risk for developing CRC.

As yet, precise estimations of the carrier rate of pathogenic *BLM* mutations in the general population have not been reported. Based on data from the Exome Aggregation Consortium^10^, the prevalence of pathogenic *BLM* mutations is 0.11%. However, in certain populations, such as in people from Ashkenazi Jewish ancestry, carrier frequencies may be as high as 1%^4^. Previous research on the risk for developing CRC in carriers with a pathologic *BLM* mutation has mainly focused on this latter group. Studies in mice have shown that the genetic background and levels of BLM protein may have an effect on the degree of tumour susceptibility^13^,^14^,^15^, which suggests that other genetic and/or environmental factors may influence the penetrance of mutant *BLM* alleles. This observation may explain why previous studies, which only focused on the Askenazi founder mutation^4^,^5^,^6^, have not been able to provide conclusive evidence of such an association.

Approximately 45% of registered Bloom syndrome patients have developed one or multiple malignancies. In approximately 12% of Bloom syndrome patients CRC was diagnosed^7^. Unfortunately, there are no reports on the incidence of CRC in family members of patients with *BLM* syndrome that have developed CRC. However, based on the incidence of CRC in Bloom syndrome patients, the penetrance for carriers will likely be even lower, and a pronounced family history for CRC in these individuals is not expected. Next to CRC, heterozygous truncating mutations in *BLM* have also been associated with an increased risk for breast cancer^3^. In two of the five families in which we identified *BLM* mutations one or more female relatives were indeed diagnosed with breast cancer. Unfortunately, we were unable to perform segregation analyses, so it therefore remains to be established whether these family members were also carriers of *BLM* mutations. However, as not all carriers of deleterious *BLM* mutations develop CRC or breast cancer, additional moderate penetrant risk factors or modifiers most likely influence cancer risk in these carriers. This latter hypothesis is in line with the idea that the risk for CRC at an early age is the result of an interplay between multiple moderate-to-low penetrant genetic and/or environmental risk factors.

Whether complete loss of *BLM* is necessary for the development of CRC in these heterozygous carriers is currently unclear. Studies in mice have suggested that monoallelic *BLM* mutations can induce tumorigenesis due to haploinsufficiency^14^, which suggests that additional loss of the wild-type allele is not strictly required for tumour initiation. In line with this, a recent study revealed that breast cancers from *BLM* mutation carriers did not show somatic inactivation of the wild-type *BLM* allele^16^. Analyses of a larger group of tumours from *BLM* mutation carriers is needed to establish the exact molecular mechanism by which heterozygous *BLM* mutations initiate tumorigenesis.

So far, deleterious *BLM* mutations have not been described in other whole-exome sequencing studies focusing on the identification of novel genetic risk factors for CRC. A possible explanation for this discordance is that these studies focused on families in which the index patient had at least one first degree relative affected by CRC or families in which the onset of the disease occurred at a later age, rather than on the group of early-onset non-familial cases that we present in this study^17^,^18^,^19^. Our study is slightly underpowered due to the limiting number of early-onset CRC cases that were available for mutation analyses. However, all comparisons between our discovery and replication cohorts, and the publically and in-house available control cohorts reveal an enrichment of deleterious *BLM* mutation in early-onset CRC cases. Validation of our findings in another non-Ashkenazi population will substantiate the role of heterozygous *BLM* mutations and the risk for CRC.

In conclusion, carriers of deleterious *BLM* mutations may have an increased risk for developing CRC at an early age, and this risk is most likely influenced by other moderate-to-low penetrant risk factors. Further knowledge on whether *BLM* acts as a classical tumour suppressor gene and/or whether other risk factors are necessary to initiate tumorigenesis, will allow for a more precise estimation of the CRC risk of heterozygous *BLM* mutation carriers.

## Materials and Methods

### Study cohorts

The discovery cohort consisted of 55 CRC cases, diagnosed at ≤45 years of age, without polyposis or a mismatch repair deficiency^20^,^21^, which were referred to the Radboud university medical center, Nijmegen, the Netherlands. The replication cohort was composed of 185 CRC cases without mismatch-repair deficiency from Nijmegen, The Netherlands and Dresden, Germany, diagnosed before the age of 50, either in the absence or presence of a family history of CRC. All participants provided written informed consent. The study was approved by the Committee on Research involving Human Subjects of the region Arnhem-Nijmegen (Commissie Mensgebonden Onderzoek (CMO) Regio Arnhem-Nijmegen; study number 2009/256), the Netherlands. The study was conducted in accordance with the approved guidelines.

### Control cohorts and enrichment analysis

The control cohort for targeted resequencing consisted of 532 irreversibly anonymized DNA samples extracted from peripheral blood from individuals with European ancestry. In addition, we retrieved all known pathogenic *BLM* mutations reported in the Bloom’s syndrome registry (http://weill.cornell.edu/bsr/)^9^ from the Exome Aggregation Consortium (ExAC)^10^, and from a cohort of 2,329 individuals of which in-house paired-end sequencing data were available with a >90-fold median coverage (Agilent V4 kit, Agilent Technologies, Santa Clara, CA, USA; Illumina HiSeq 2500; Illumina, San Diego, CA, USA). These latter exomes were sequenced as a part of routine diagnostic testing for multiple conditions, excluding hereditary cancers, at the department of Human Genetics, Nijmegen, the Netherlands^21^, and the data were analysed anonymously. We calculated that our replication study, with the above-mentioned CRC and control sample sizes, would result in a power of 70%. Power calculations and the Fisher’s exact test to calculate odds ratio’s (OR) and significance of differences between the CRC cohort and control cohorts were performed using the statistical software package R (http://www.R-project.org/).

### Targeted resequencing by molecular inversion probes

Molecular inversion probes (MIPs) were designed as described by the procedure of O’Roak *et al.*^8^ against the coding region and at least 20 nucleotide intronic sequences up- and downstream of each exon of the *BLM* gene. Aliquots of each 70-mer oligonucleotide probe (Integrated DNA Technologies, Coralville, IA, USA) were pooled at equimolar ratios and the 5′-end of each probe was phosphorylated using T4 polynucleotide Kinase (New England Biolabs, Ipswich, MA, USA). Genomic DNA was prepared from peripheral blood cells or paraffin embedded formalin-fixed (tumour or normal colonic) tissue using standard procedures. MIP-based target enrichment was performed as described previously with minor modifications^8^,^22^. Briefly, 100 ng of genomic DNA was used to capture the target regions. DNA, the phosphorylated MIP pool, dNTPs, polymerase and ligase were pooled, denatured for 10 min at 95 °C and MIP capture was performed for 22 h at 60 °C. After an exonuclease treatment a PCR was performed to amplify the captured material together with forward and barcoded reverse primers suitable for Ion Torrent PGM sequencing: 5′-CCTCTCTATGGGCAGTCGGTGATATCGGGAAGCTGAAG-3′ and 5′- CCATCTCATCCCTGCGTGTCTCCGACTCAGXXXXXXXXACGATATCCGACGGTAGTGT-3′ (XXXXXXXX represents the 8 bp barcode). The resulting PCR products were pooled and purified twice using Ampure XP beads (Agencourt, Beckman Coulter, Pasadena, CA, USA) according to the manufacturer’s protocol. This final library was diluted for use in a 200 bp amplification run on an OneTouch emulsion PCR system (Life Technologies, Carlsbad, CA, USA). The resulting Ion spheres were run on an Ion Torrent PGM sequencing platform (Life Technologies). Reads were aligned to UCSC human genome assembly hg19 and called and annotated using SequencePilot (JSI medical systems, Ettenheim, Germany). All variants called with a minimal variant percentage of 20% and a minor allele frequency of <1% in dbSNP138 were selected for further analyses. Frameshift variants adjacent to homopolymeric nucleotide repeats that occurred in ≥10% of the samples were excluded as these were considered platform-specific artefacts. Variants predicted to be deleterious were validated using Sanger sequencing.

### LOH and copy-number analyses

Locus-specific loss of heterozygosity (LOH) analyses were performed using DNA extracted from tumour material and adjacent normal tissue. The respective DNA fractions were used to perform PCRs generating small amplicons covering the patient-specific mutations, which were sequenced by Sanger sequencing (primers and conditions available upon request). Genome-wide SNP array analysis of tumour DNA was performed using the OncoScan FFPE Express service (Affymetrix, Santa Clara, CA, USA). The SNP array data were analysed using the Nexus Copy Number software package (Biodiscovery, Hawthorne, CA, USA).

## Additional Information

**How to cite this article**: de Voer, R. M. *et al.* Deleterious Germline *BLM* Mutations and the Risk for Early-onset Colorectal Cancer. *Sci. Rep.* **5**, 14060; doi: 10.1038/srep14060 (2015).

## Supplementary Material

Supplementary Information

## Figures and Tables

**Table 1:** Deleterious *BLM* mutations in patients with early-onset colorectal cancer were identified using whole-exome (discovery cohort) or targeted (replication cohort) resequencing.

**Case**	**Nucleotide change**	**Mutation**	**rs-number**	**Sex**	**Age of onset**	**Familial history for cancer**^a^	**Tumour available**	**2nd hit**
Discovery cohort (*n* = 55)								
P008	c.3558 + 1G > T	Splicing	rs148969222	F	29	2CRC (2x)	No	N/A
P034	c.2695C > T	p.Arg899*	–	M	37	1CRC (2x), 2CRC (1x)	Yes	Tumour LOH
Replication cohort (*n* = 185)								
RC001	c.98 + 1G > A	Splicing	–	F	45	Multiple 1^st^ and 2^nd^ degree family members with cancer	Yes	No^b^
RC002	C.1642C > T	p.Gln548*	rs200389141	M	38	2CRC (2x)	Yes	No
RC003	c.2695C > T	p.Arg899*	–	F	43	2CRC (6x)	Yes	No^b^

^a^The number preceding the type of cancer indicates the familial relation, e.g. 1, first-degree relative; 2, second-degree relative. F, female; M, male; CRC, colorectal cancer; LOH, loss of heterozygosity; N/A, not available.

^b^Samples were only analysed for tumour LOH.

**Table 2:** Enrichment analyses for known deleterious *BLM* mutations in early-onset CRC cases compared to a cohort of population-matched controls in in-house and public databases.

	**Control cohort (1/532)**	**In-house control exomes**^a^ **(2/2,329)**	**ExAC**^b^ **(71/61,486)**
	OR: 8.67	OR: 18.99	OR: 14.15
Replication cohort (3/185)	95% CI, 0.69–455.36	95% CI, 2.17–227.56	95% CI, 2.85–43.37
	*P* = 0.05516	*P* = 0.003534	*P* = 0.001482

^a^In-house data set of sequenced exomes;

^b^ExAC, Exome Aggregation consortium^10^;

Abbreviations: OR, odds ratio; CI, confidence interval.
