# Supplementary material for: Deleterious Germline BLM Mutations and the Risk for Early-onset Colorectal Cancer
Source: Sci Rep. 2015 Sep 11;5:14060. doi: 10.1038/srep14060 (PMC4566092; doi:10.1038/srep14060)
Supplement: Supplementary Information [file srep14060-s1.pdf]

Supplementary online material to:

Deleterious Germline *BLM* Mutations and the Risk for Early-onset Colorectal Cancer

Richarda M. de Voer[1,*], Marc-Manuel Hahn[1,*], Arjen R. Mensenkamp[1*], Alexander Hoischen[1], Christian Gilissen[1], Arjen Henkes[1], Liesbeth Spruijt[1], Wendy A. van Zelst-Stams[1], C. Marleen Kets[1], Eugene T. Verwiel[1], Iris D. Nagtegaal[2], Hans K. Schackert[3], Ad Geurts van Kessel[1], Nicoline Hoogerbrugge[1], Marjolijn J.L. Ligtenberg[1,2] and Roland P. Kuiper[1#]

[1]*Department of Human Genetics, Radboud university medical center, Geert Grooteplein 10, 6525 GA, Nijmegen, The Netherlands;*
[2]*Department of Pathology, Radboud university medical center, Geert Grooteplein 10, 6525 GA, Nijmegen, The Netherlands;*
[3]*Department of Surgical Research, University Hospital Carl Gustav Carus, Technische Universität Dresden, Fetscherstraße 74, 01307, Dresden, Germany*;
*These authors contributed equally to the presented work

[#]**Corresponding author**
Roland P. Kuiper; Department of Human Genetics; Radboud university medical center; PO Box 9101; 6500 HB Nijmegen; The Netherlands Tel.: +31-24-3610868; Email: Roland.Kuiper@radboudumc.nl

Supplementary Figures

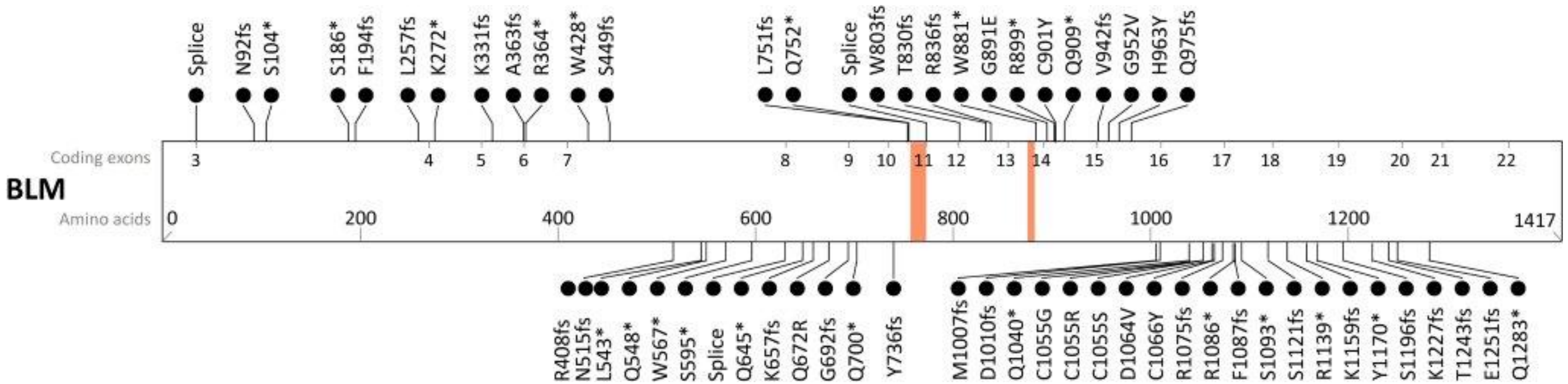

Supplementary figure 1: Distribution of known pathogenic *BLM* mutations as reported in the Bloom's Syndrome Registry (http://weill.cornell.edu/bsr/). Regions that were not properly covered by our molecular inversion probe design (<10-fold) are indicated in orange. These regions, located in exons 10 and 13, represent ~ 2.6% of the total coding sequence and they do not overlap with known pathogenic mutations.

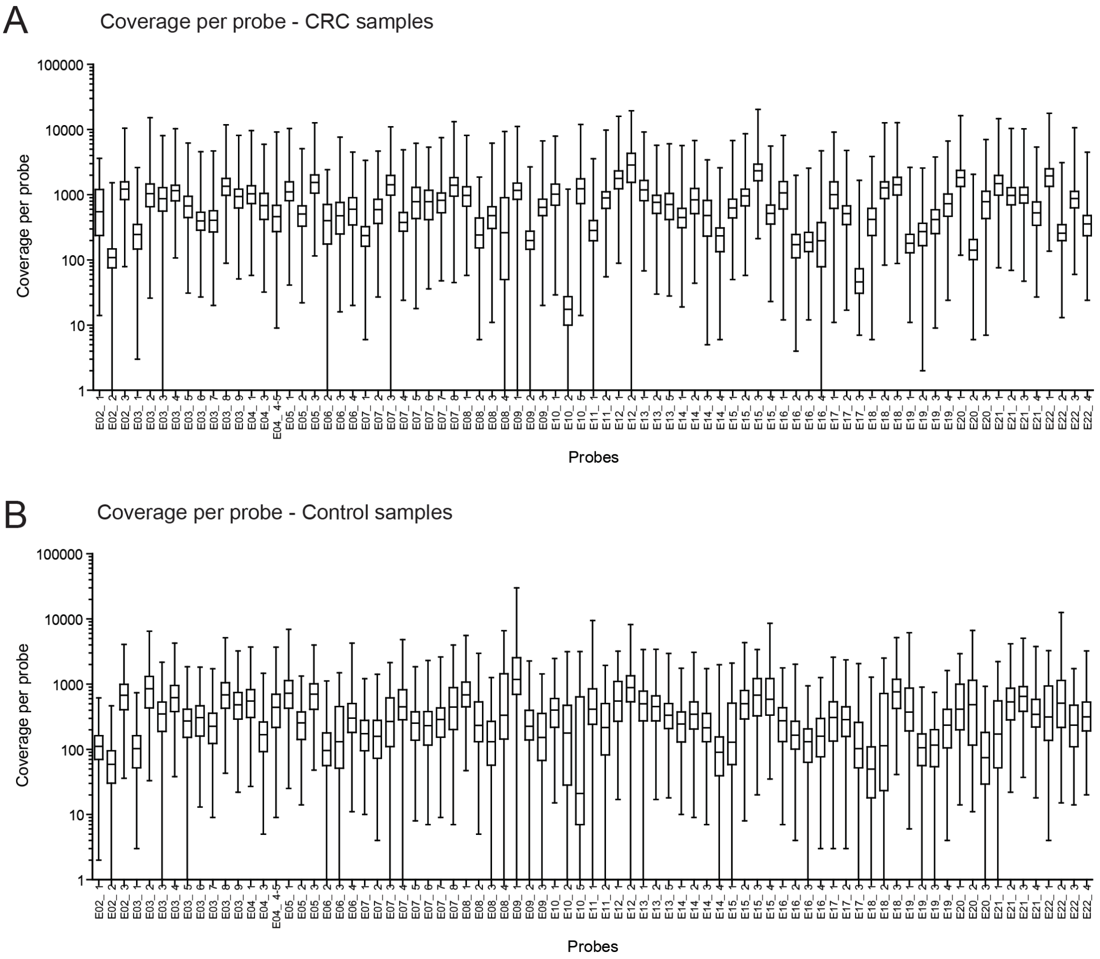

Supplementary figure 2: Box plots of the coverage per molecular inversion probe. (**A**) Coverage per probe for the CRC samples (*n*=185). (**B**) Coverage per probe for the population-matched control cohort (*n*=532).

A

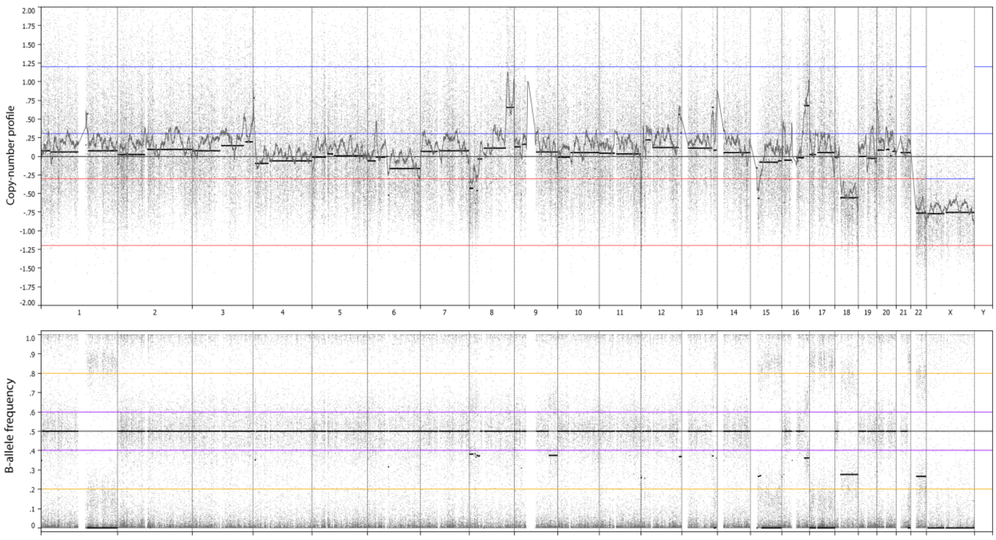

B

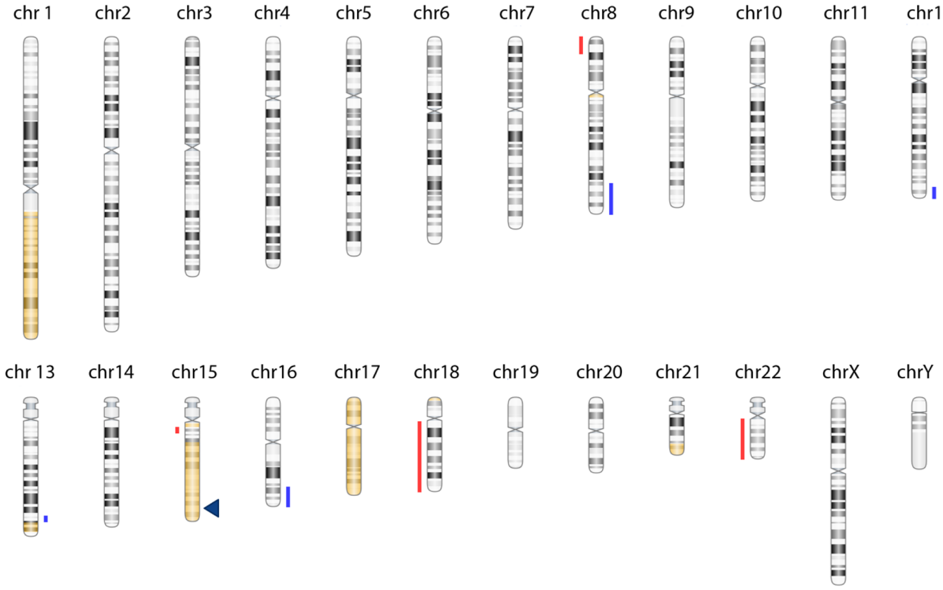

Supplementary figure 3: Overview of the genomic profiling of the tumor of individual P034. Analysis was performed using Nexus Copy Number, version 6. To accommodate for a noisy signal a smoothing of 0.8 was applied. Significance thresholds were set to $1x10^{-15}$, allelic balance calls ignored and minimal length of loss of heterozygosity (LOH) areas defined as greater than 1 Mb to assure genomic significance. (**A**) The top graph showing the copy number profile. Note the copy number loss of 8pter, 18 and 22 and the gain of of 8qter and 16qter (blue bars). For chromosomes 15, 17, and 1q the B-allele frequency plot shows a copy-neutral loss-of-heterozygosity. (**B**) Diagram showing the genomic profiling results of the tumor of individual P034 as determined by Affymetrix Oncoscan array analysis. Copy-neutral LOH of chromosomes 15, 17, and 1q is depicted by yellow shading, deletion of 8pter, 18 and 22 by red bars, and duplication of 8qter and 16qter by blue bars. The location of the *BLM* locus on chromosome 15 is indicated by an arrowhead.
